# Prediction of Preeclampsia Using Machine Learning: A Systematic Review

**DOI:** 10.7759/cureus.76095

**Published:** 2024-12-20

**Authors:** Vinayak Malik, Neha Agrawal, Sonal Prasad, Sukriti Talwar, Ritu Khatuja, Sandhya Jain, Nidhi Prabha Sehgal, Neeru Malik, Jeewant Khatuja, Nikita Madan

**Affiliations:** 1 Computer Science, University of Wisconsin, Madison, USA; 2 Obstetrics and Gynecology, Dr. Baba Saheb Ambedkar Hospital and Medical College, New Delhi, IND; 3 Computer Science, Delhi Technological University, New Delhi, IND; 4 Anesthesiology and Critical Care, Dr. Baba Saheb Ambedkar Hospital and Medical College, New Delhi, IND; 5 Obstetrics and Gynecology, Dr. Baba Saheb Ambedkar Medical College and Hospital, New Delhi, IND; 6 Automation and Robotics, University School of Automation and Robotics, Guru Gobind Singh Indraprastha University, New Delhi, IND; 7 Obstetrics and Gynecology, ESI Hospital and Postgraduate Institute of Medical Sciences and Research (PGIMER) Basaidarapur, New Delhi, IND

**Keywords:** artificial intelligence, deep learning, machine learning, preeclampsia, risk of bias, systematic review

## Abstract

Preeclampsia is one of the leading causes of maternal and perinatal morbidity and mortality. Early prediction is the need of the hour so that interventions like aspirin prophylaxis can be started. Nowadays, machine learning (ML) is increasingly being used to predict the disease and its prognosis. This review explores the methodologies, predictors, and performance of ML models for preeclampsia prediction, emphasizing their comparative advantages, challenges, and clinical applicability.

We conducted a systematic search of databases including PubMed, Cochrane, and Scopus for studies published in the last 10 years using terms such as “preeclampsia”, “risk factors”, “machine learning”, “artificial intelligence”, and “deep learning”. Words and phrases were selected using MeSH, a controlled vocabulary. Appropriate articles were selected using Boolean operators “OR” and “AND”. The database search yielded 325 records. After removing duplicates and non-English articles, and completing a title and abstract search 55 research articles were assessed for eligibility of which 11 were included in this review. The risk of bias was found to be high in three of the studies and low in the rest. Clinicodemographic characteristics, laboratory reports, Doppler ultrasound, and some innovative ones like genotypic data and fundal images were predictors used to train ML models. More than ten different ML models were used in the 11 studies from diverse countries like the United States, the United Kingdom, China, and Korea. The area under the curve varied from 0.76 to 0.97.

ML algorithms such as extreme gradient boosting (XGBoost), random forest, and neural networks consistently demonstrated superior predictive accuracy Non-interpretable or black box ML models may not find clinical application on ethical grounds. The future of preeclampsia prediction using ML lies in balancing model performance with interpretability. Human oversight remains indispensable in implementing and interpreting these models to achieve better maternal outcomes. Further research and validation across diverse populations are critical to establishing the universal applicability of these promising ML-based approaches.

## Introduction and background

The aim of the United Nations Sustainable Development Goal (SDG) 3.1 is to reduce the world maternal mortality ratio to less than 70 per 100,000 live births by 2030 [1]. The underdeveloped countries bear the maximum burden of maternal mortality, particularly in sub-Saharan Africa (66%, 201,000 deaths) and southern Asia (22%, 66,000 deaths) [2]. Preeclampsia is one of the leading causes of maternal morbidity and mortality, leading to placental abruption and eclampsia [3]. The incidence of preeclampsia in less-developed countries varies from 4.0% to 12.3% [3-7] and it is responsible for 12 % of maternal mortality [8]. Early identification of women at risk is critical for implementing preventive strategies, including aspirin prophylaxis, lifestyle modifications, and intensified monitoring. Most pregnancy hypertension estimates in less-developed countries are from cross-sectional hospital surveys, but due to multiple pathogenesis, various risk factors, and numerous phenotypes, early predictions of preeclampsia are difficult.

Various predictive models are used to stratify women at risk of preeclampsia. Conventional general programming algorithms like logistic regression (LR) generate outputs based on the input data and the rules provided [9]. It was widely accepted due to its simplicity and interpretability. Variables like clinical and laboratory data of patients were dealt with well within statistical learning as they automatically select the most informative character [10]. However, LR generates a linear relationship between predictors and outcomes, limiting its capacity to capture complex and nonlinear patterns inherent in large and diverse datasets. Due to recent advances in computer science artificial intelligence has been increasingly used in obstetrics and gynecology departments [11,12]. Based on input and output data, artificial intelligence can create a pattern [13].

Machine learning (ML) offers an alternative approach to predictive models. ML encompasses a range of algorithms, including random forests, support vector machines, and deep learning, that excel at identifying intricate patterns in data. ML can handle high-dimensional datasets, account for interactions between predictors, and automatically learn optimal feature representations. Studies suggest that ML models may outperform LR in terms of predictive accuracy, particularly in the presence of nonlinear relationships and large, complex datasets. In the medical field, pattern recognition and prediction performance of artificial intelligence are taken into consideration [14]. For early and adequate identification of women at risk of preeclampsia a systematic review of existing predictive models was needed. This allows us to determine whether the existing predictive model is suitable for immediate use. This also identifies reassessments that perform well internally but, before being considered for clinical use, need external validation on an independent cohort [15]. Adding a new model to help prevent preeclampsia demands more time and resources. Thus, this approach is more efficient [15]. However, the increased complexity of ML models introduces challenges related to interpretability, computational requirements, and integration into clinical workflows. 

This review seeks to analyze and compile a thorough evaluation of the ML models used in predicting preeclampsia. We evaluated the sample size, geographical area of study, predictors used to train the ML model, methodological frameworks, predictive performance, and clinical relevance while discussing the advantages and limitations of each. By examining the current literature, this systematic review highlights the potential of ML in advancing preeclampsia prediction. 

## Review

The present review was reported using Preferred Reporting Items for Systematic Reviews and Meta-Analyses (PRISMA) 2020 guidelines [16]. The Prediction Model Risk of Bias Assessment Tool (PROBAST) was used to assess the risk of bias (ROB) and applicability of each of the 11 studies reviewed [17].

Inclusion and exclusion criteria

Studies on preeclampsia prediction using ML that reported performance metrics such as Area Under the Curve (AUC), sensitivity, and specificity were included. Studies that lacked methodological clarity or performance metrics, as well as non-English studies, were excluded.

Data collection and ROB assessment 

The following data were extracted: 

(1) Demographic information (data sources, participants, and sample size) 

(2) Predictors used 

(3) The types of predictive model algorithms used and the one that performed best 

(4) Prediction results, such as AUC, sensitivity, and specificity 

(5) The features used to train the ML models 

**Figure 1:**
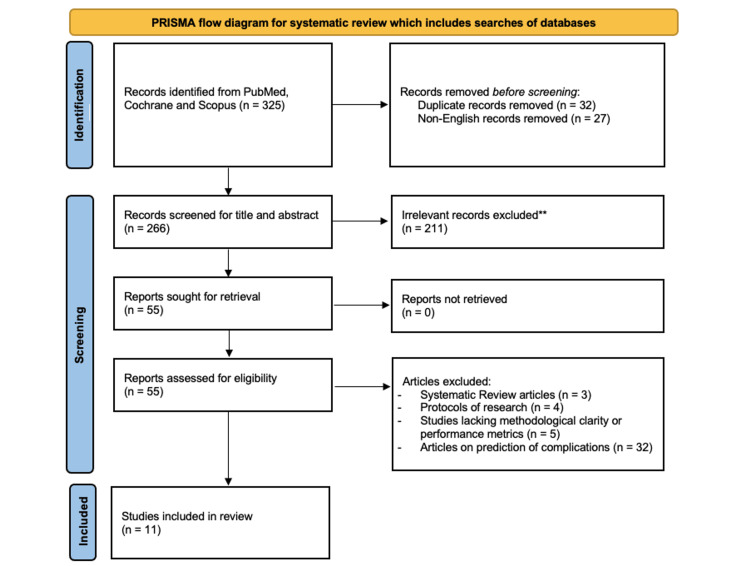
PRISMA 2020 flow diagram for new systematic reviews that included searches of databases. PRISMA, Preferred Reporting Items for Systematic Reviews and Meta-Analyses

We conducted a systematic search of databases, including PubMed, Cochrane, and Scopus, for studies published in the last 10 years using terms such as “preeclampsia”, “risk factors”, “machine learning”, “artificial intelligence”, and “deep learning”. Words and phrases were selected using MeSH, a controlled vocabulary. Appropriate articles were selected using Boolean operators “OR” and “AND”. Hand-searching of reference lists of the identified articles was also done to ensure that all documents were retrieved. All the duplicates were eliminated. The articles which were irrelevant and non-English articles were excluded from the study. Review articles were also excluded. As per the predefined eligibility criteria, two experienced researchers screened the titles and abstracts and then the full texts of potentially eligible studies to be included in this study. The process of selection of relevant studies was documented using a PRISMA flow diagram (Figure 1) [16].

PROBAST was used to assess the ROB and applicability of each of the 11 studies reviewed (Table 1) [17].

**Table 1:** ROB and ROA using PROBAST. ROB, risk of bias; ROA, risk of applicability; PROBAST, Prediction Model Risk of Bias Assessment Tool

S. nNo.	Study and year	Participants (ROB)	Predictors (ROB)	Outcome (ROB)	Analysis (ROB)	Overall ROB	Participants (ROA)	Predictors (ROA)	Outcome (ROA)	Overall ROA
1	Jhee et al., 2019 [18]	Low	Low	Low	Low	Low	Low	Low	Low	Low
2	Marić et al., 2020 [19]	Low	Low	High	Low	Low	Low	Low	High	Low
3	Li et al., 2021 [20]	Low	Low	Low	Low	Low	Low	Low	Low	Low
4	Ansbacher-Feldman et al., 2022 [21]	Low	Low	Low	Low	Low	Low	Low	Low	Low
5	Liu et al., 2022 [22]	Low	Low	Low	Low	Low	Low	Low	Low	Low
6	Eberhard et al., 2023 [23]	Low	Low	Low	Low	Low	Low	Low	Low	Low
7	Kovacheva et al., 2023 [24]	Low	Low	Low	High	High	Low	Low	Low	Low
8	Tiruneh et al., 2024 [25]	Low	Low	Low	Low	Low	Low	Low	Low	Low
9	Wang et al., 2024 [26]	Low	Low	Low	Low	Low	Low	Low	Low	Low
10	Khalil et al., 2024 [27]	Low	High	Low	Low	High	Low	High	Low	High
11	Zhou et al., 2024 [28]	Low	High	Low	Low	High	Low	High	Low	High

PROBAST was developed through a consensus process involving a group of experts in the field [17]. PROBAST consists of 4 domains containing 20 signaling questions to facilitate ROB assessment. The 4 domains are participants, predictors, outcome, and analysis [17]. An overall judgment of “low ROB” or “low concern regarding applicability ” was said when a prediction model evaluation is judged as low on all domains relating to bias and applicability If an evaluation is judged as high for at least one domain, it should be judged as having “high ROB” or “high concern regarding applicability.” An “unclear ROB” or “unclear concern regarding applicability” was said if the prediction model evaluation was unclear in one or more domains and was rated as low in the remaining domains [17].

This systematic review was undertaken to assess the commonly used ML approaches for predicting preeclampsia as a small contribution to risk prediction using the artificial intelligence (AI) field in 11 ML studies. A comparative evaluation of the performance and accuracy of the various ML studies reviewed is depicted (Table 2).

**Table 2:** Comparative evaluation of studies. EHR, electronic health record; UtA-Doppler, uterine artery Doppler; MAP, mean arterial pressure; AUC, area under the curve; ML, machine learning; GB, gradient boost; LR, logistic regression; DT, decision tree; DNN, deep neural network; NB, naive Bayes; RF, random forest; EN, elastic net; SGB, stochastic gradient boosting; AdaBoost, adaptive boosting; MLP, multi-layer perceptron; GBDT, gradient boosting decision tree; XGBoost, extreme gradient boosting; SVM, support vector machines; CatBoost, category boosting; LightGBM, light gradient boosted machine; CNN, convolutional neural network; DL, deep learning; NR, not reported; MARS, multivariate adaptive regression splines

S. no.	Study and year	Country	Data set Size	Type of study	Predictors	Algorithms	Best-performing algorithm	Performance (AUC)	Sensitivity/Specificity	Key findings
1	Jhee et al. (2019) [18]	Korea	11,006	Prospective cohort	Clinicodemographic characteristics, laboratory parameters	LR, DT, NB, SVM, RF, SGB	SGB	0.92	Sensitivity: 60%, Specificity: 99%, Detection rate: 0.77, Accuracy: 0.97	Developed models for late-onset preeclampsia using early second-trimester data. Used pattern recognition and cluster analysis to find influential predictors.
2	Marić et al. (2020) [19]	US, single center	16,370	Retrospective cohort	Clinicodemographic characteristics, laboratory parameters (EHR data)	LR, EN, GB	EN	0.89	Sensitivity: 45%, Specificity: 81%, TPR : 72.3%, FPR: 8.8%	Early prediction of preeclampsia
3	Li et al. (2021) [20]	China	5243	Retrospective	Clinicodemographic characteristics (questionnaire based)	LR, RF, SVM, XGBoost	XGBoost	0.97	Sensitivity: 81%, Specificity: 83%	Questionnaire-based data, less missing data
4	Ansbacher-Feldman et al. (2022) [21]	United Kingdom	60,789	Prospective, non-interventional	First-trimester maternal characteristics, UtA-Doppler, MAP, biomarkers	ML ensemble with neural network algorithms	Not specified	0.91	Sensitivity: 75%, Specificity: 82%	Combined biomarkers and maternal history for early-onset preeclampsia prediction
5	Liu et al. (2022) [22]	China	11,472	Retrospective cohort single center	Clinicodemographic characteristics	DNN, LR, SVM, DT, RF	RF	0.86	Sensitivity: 74%, Specificity: 82%	Retrospective cohort study demonstrating moderate predictive accuracy
6	Eberhard et al. (2023) [23]	United States	120,752	Retrospective multicenter	Clinicodemographic characteristics, laboratory data.( EHR)	XGBoost, EN, RF, linear regression	XGBoost	0.76 at 14 weeks		Multistage prediction using ML on EHR, AUC at admission is 0.91.
7	Kovacheva et al. (2023) [24]	United States	1125	Retrospective cohort	Genetic and clinical predictors	Logistic regression, XGBoost	XGBoost	0.91	Sensitivity: 85%, Specificity: 87%	In both early and late pregnancies, machine learning models performed better than logistic regression models; XGBoost in late pregnancy was the most predictive.
8	Tiruneh et al. (2024) [25]	Australia	48,250	Retrospective cohort	Clinicodemographic characteristics (EHR)	RF, XGBoost, SVM, MARS, NB, neural network	RF	0.84	Sensitivity: 80%, Specificity: 83%	Leveraged routinely collected data to improve prediction accuracy
9	Wang et al. (2024) [26]	China	30,000	Retrospective cohort study	Clinicodemographic characteristics (EHR)	AdaBoost, RF, MLP, GBDT, XGBoost, LR, SVM, CatBoost, LightGBM	AdaBoost	0.88	Sensitivity: 72%, Specificity: 90%	Very high specificity and moderate sensitivity Very high negative predictive value
10	Khalil et al(2024) [27]	Multicenter, multi-country	17,520	Prospective observational	Clinicodemographic characteristics, cell-free DNA	LR, neural network	Similar performance	0.8	Sensitivity: 0.68	Secondary analysis of a prospective, multicenter, observational prenatal cell-free DNA screening study (SMART) included singleton pregnancies with known pregnancy outcomes.
11	Zhou et al. (2024) [28]	China	1183	Prospective cohort	Retinal fundus images	DL	CNN	0.88	Sensitivity: 0.72, Specificity: 0.93	Deep learning algorithms based on retinal fundus images are noninvasive, low-cost, and do not require expert technicians to simplify the prediction process and save resources.

Discussion

It is estimated that 1.3 million women died from maternal causes over the last two decades in India. Preeclampsia is a leading cause of maternal mortality in India and is responsible for at least 7% of deaths, third only behind hemorrhage and sepsis [29]. The pathogenesis of preeclampsia is only partially understood due to its multiple phenotypes. Preeclampsia is caused by endothelial dysfunction, impaired trophoblast invasion, incomplete spiral artery remodeling at the beginning of pregnancy, immunologic aberrations, and multiple genetic components [30]. Due to multiple pathogenesis and varied clinical presentation, screening and prediction of the severity of preeclampsia remains controversial. Aspirin started in a dose of 150 mg per day at 11-14 weeks of gestation can be used in primary prevention of preeclampsia in high-risk women identified by screening tests [31]. 

Hence, the need of the hour is accurate and easily implementable screening to identify women at risk of preeclampsia. For the immediate application of prophylactic measures, the National Institute for Health and Clinical Excellence (NICE 2020) guidelines have classified, the risk factors for preeclampsia as “moderate risk” and “high risk” [32]. If a woman has a single high-risk factor or two moderate risk factors, prophylactically aspirin has to be started before the 16th week of gestation and the patient should follow up in a specialized center [32]. The American College of Obstetricians and Gynecologists (ACOG) 2018 categorizes risk factors of preeclampsia into “low risk,” “moderate risk,” and “high risk [33]. The sensitivity and specificity of preeclampsia based on NICE guidelines is 77% and 54%, respectively. Based on an incidence of preeclampsia of 4% the positive predictive values of NICE guidelines were estimated at only 7% [34].

A large number of first-trimester prediction models have failed external validation. The Fetal Medicine Foundation (FMF) first-trimester prediction model, a competing risk model based on the Bayesian principle, combines maternal factors, mean arterial pressure, uterine artery pulsatility index, and biochemical markers like serum placental-induced growth factor (PIGF). The FMF algorithm has a sensitivity of 35%-77% and a specificity of over 70% [35]. Traditional methods screened preeclampsia by maternal risk factors alone so the FMF model of screening is superior. However, in resource-constraint setups in developing nations, the FMF prediction model is of limited value as it requires Doppler expertise and expensive biochemical marker testing. 

It is indeed ironic that in spite of the large body of research in the prediction of preeclampsia and the availability of the low-cost and safe drug, Aspirin, for prevention, preeclampsia continues to be seen as a major killer in pregnant women across the developing world. Recent literature suggests the lack of implementation of these models in clinical settings is a major reason [36]. 

Routinely collected clinicodemographic information contains risk factors associated with preeclampsia, such as family or personal history of preeclampsia, multiple pregnancies, nulliparity, raised blood pressure at antenatal visits, increased body mass index (BMI) before or during pregnancy, advanced maternal age, interval of 10 years or more between pregnancies, autoimmune disease, renal disease, diabetes, sedentary lifestyle, use of condoms, change of paternity, smoking and psychosocial stress. 

With the advent of AI, there has been an increasing interest in the use of large line listing patient data available in healthcare settings to create high-performance clinical screening ML algorithms for disease prediction. This systematic review was undertaken to assess the commonly used ML approaches for predicting preeclampsia as a small contribution to risk prediction using the AI field.

Jhee et al. in a study from Korea retrieved clinicodemographic and laboratory data of 11,006 women from the early second trimester to 34 weeks [18]. These data were divided randomly into two sets 70:30 for training the ML models for prediction and validation, respectively. The study outcome measure was late-onset preeclampsia after 34 weeks. Using pattern recognition and cluster analysis, the influence of repeated measured variables on prediction was evaluated. The AUC for the support vector machine (SVM), decision tree model, random forest algorithm, naïve Bayes classification, stochastic gradient boosting method, and logistic regression models were 0.573, 0.857, 0.894, 0.776, 0.924, and 0.806, respectively. The stochastic gradient boosting model performed the best in prediction accuracy and false positive rate, with values of 0.973 and 0.009, respectively. The absence of late first-trimester prediction modeling when intervention with Aspirin is optimal was not available limiting the usefulness of the model.

Maric et al. conducted a retrospective cohort study using data at <16 weeks gestational age from 16,370 births in a single center in California [19]. Maternal characteristics, medical history, routine prenatal laboratory results, and medication intake were among the 67 variables that were considered in the models. These variables were fitted into ML models for the prediction of early-onset preeclampsia. Elastic net algorithm performed best for early-onset preeclampsia. It depicted an area under the curve of 0.89, a true-positive rate of 72.3%, and a false-positive rate of 8.8%. A main limitation of this study was the substantial amount of missing data. 

Li et al. included a total of 5,243 pregnant women in a retrospective study from China using questionnaire-based data collection from electronic health records (EHR) [20]. clinical and laboratory parameters routinely available at the first visit in antenatal care were collected by manual chart review. Diagnostic criteria of preeclampsia as per International Society for the Study of Hypertension in Pregnancy (ISSHP) guidelines [37] were strictly followed and the outcome measures were preterm, term, and late-onset preeclampsia. A prediction model was constructed using random forest (RF), support vector machine (SVM), extreme gradient boosting (XGBoost), and LR. The XGBoost model had the best prediction performance (c, recall = 0.789, f1_score = 0.571, auROC = 0.955). The most predictive feature of the development of preeclampsia was fasting plasma glucose, followed by mean blood pressure and BMI.

Ansbacher-Feldman et al. used EHR data from two maternity hospitals in the United Kingdom for a prospective non-interventional screening for preeclampsia at 11-13 weeks gestation [21]. The data were divided into three subsets. The first set, including 30,437 subjects, was used to develop, the second set of 10,000 subjects to optimize the machine-learning hyper-parameters, and the third set of 20,352 subjects were coded and used for model validation. The risk of preeclampsia and preterm preeclampsia was predicted by demographic characteristics, medical history of the patient, and biomarker values using an artificial neural network. Mean arterial blood pressure (MAP), uterine artery pulsatility index (UtA-PI), pregnancy-associated plasma protein-A, and placental growth factor (PlGF) were biomarkers included in the study. The impact of taking aspirin prophylactically was also added. Shapley Additive exPlanations (SHAP) is an explainable artificial AI method used to determine the importance of each included feature/variable in the ML approach. The SHAP plot is used to describe the marginal contributions of input features to the output of the ML model at the instance level [38]. When screening by maternal factors only, at a false positive rate of 10%, the detection rate for preterm preeclampsia vs no preeclampsia was 53.3%. With the addition of biomarkers, the detection rate increased to 75.3% and 0.909, respectively. This model was externally validated in a large study in Spain [39] with a detection rate of preterm preeclampsia of 77.8% with an AUC of 0.91. It is still early to say if the models derived using ML have better applicability in populations away from the ones they were derived from as compared to classical regression models. More research in this direction may lead to valuable insight. 

Eberhard et al. in a study from the United Kingdom extracted data from 12,075 patients to perform a multistage prediction using ML on routinely available clinical and laboratory data [23]. The XGBoost model had the highest AUC. As pregnancy progressed and gestational weeks increased, the accuracy of the model gradually improved, starting from an AUC of 0.76 and reaching 0.91 before delivery, demonstrating the highest predictive value. However, since intervention is possible only if preeclampsia is predicted early so the high accuracy close to delivery has no clinical relevance. 

Kovacheva et al. used genotyping data available from 1125 pregnancies and hypothesized that the simultaneous use of clinical risk factors and a hypertension genetic risk score ie, polygenic risk score [PRS] could improve preeclampsia risk prediction for pregnant individuals [24]. They developed logistic regression models, which perform well with binary outcomes, and XGBoost ML models, which have high interpretability and perform well in structured data from EHR. They developed a clinical XGBoost model, which had an AUC of 0.74. Adding the systolic blood pressure (SBP) PRS did not result in statistically different predictive abilities (*P *= 0.11). The most predictive variables in the model (determined using the Shapley interpretability method) were blood pressure, maternal age, and history of preeclampsia in a prior pregnancy. 

In a large prospective cohort study [25] using the public health network data set of 48,250 women from 78 diverse nationalities in Australia, maternal demographic, clinical, obstetric characteristics and birth outcomes were collected prospectively to predict preeclampsia using common ML models, namely random forest, NB, gradient boosting, XGBoost, decision tree, SVM, MARS, neural network, CatBoost classifier, and multivariable logistic regression model. The study was reported according to the TRIPOD- AI (Transparent Reporting of a multivariable prediction model for Individual Prognosis or Diagnosis) - AI: artificial intelligence checklist [35]. Shapley values were calculated to evaluate the contribution of each parameter to the prediction of risk [38]. XGBoost, gradient boosting, logistic regression, and MARS were the four models that achieved good discrimination performance (AUC ≥ 0.70) in the testing dataset. The best-performing model was random forest with AUC ≥ 0.80. The feature variables arranged in descending order of their marginal average contribution to the model were: nulliparity, pre-pregnancy BMI, previous pregnancy with prior preeclampsia, maternal age, family history of hypertension, and preexisting hypertension, and diabetes mellitus (DM). These have a significant contribution to the high risk of preeclampsia. 

Wang et al. performed a retrospective cohort in 25,709 pregnancies by developing an early screening model and validated it in a validation cohort of 1760 pregnancies [26]. On augmented data, 10 ML models were simultaneously trained. The AdaBoost model, utilizing 16 predictors, was the best predicting model, achieving an area under the receiver operating characteristic curve of 0.80 and a sensitivity of 0.51.

Khalil et al. reported that, apart from traditional predictors, preeclampsia was associated with higher total cell-free DNA (median, 362.3 vs. 339.0 copies/mL cell-free DNA; *P* < 0.001) and lower fetal fraction (median, 7.5% vs. 9.4%; *P* < 0.001) [27]. Routinely available patient characteristics and cell-free DNA (cfDNA), fetal fraction (FF), and cell-free RNA (cfRNA) markers can be used to predict preeclampsia. A neural network model based on cfDNA and FF had a sensitivity of 58.4%, while a fully convolutional dense network (FcDN) architecture model based on cfRNA achieved an accuracy of 0.95.

Zhou et al. evaluated the use of deep learning on retinal fundus images for predicting preeclampsia [28]. They demonstrated strong predictive values, with AUCs of 0.845 for preeclampsia. The convolutional neural network model to process fundus retinal images and derive the predicted probability of hypertension, defining it as the *fundus score* looks promising in preeclampsia prediction.

While the earlier studies on preeclampsia prediction used the known clinicodemographic, Doppler, and biochemical predictors available as EHR to make ML prediction models, there is now an increasing interest in using EHR data like fundal images, genotyping, etc., to predict preeclampsia. While there is no doubt that ML-based models have higher accuracy and can analyze complex, nonlinear data in seconds, it is not without shortcomings. The black-box nature of high-accuracy ML models limits their clinical application.

Model explanation techniques like SHAP or Local Interpretable Model agnostic Explanation (LIME) are promising tools that are increasingly being deployed and represent two prominent interactive techniques for model behavior [35]. SHAP is a commonly used approach that quantifies the individual contribution of a feature value (Shapley value) to the difference between the actual and the average prediction of the model, detailed as relative distribution among features [39]. LIME can be used to explain how individual features lead to prediction probabilities by approximating it locally with an interpretable model [40].

The primary limitation of this study is that it only includes research from developed countries such as the United States, Australia, the United Kingdom, and Korea, along with a developing country like China. It does not account for countries like India and those in Africa, where preeclampsia rates are high and healthcare infrastructure is often inadequate. Most of the studies used EHRs, which may have missing data resulting in inaccuracies in prediction by the ML models. The studies reviewed diverse datasets, differing in sample size, quality, and geographic origin. This variability makes it difficult to draw direct comparisons or make universal conclusions about the effectiveness of ML models. While models like those by Ansbacher-Feldman et al. [21] were externally validated, most others were not, limiting their applicability to different populations. Lastly, the exclusion of papers in languages other than English could be seen as a limitation.

## Conclusions

ML algorithms such as XGBoost, random forest, and neural networks consistently outperform traditional prediction methods, offering higher accuracy and the ability to analyze complex, nonlinear data. However, several challenges limit the clinical applicability of these models, including the black-box nature of many algorithms, variability in data sources, lack of external validation, and geographic bias in the studies. Non-interpretable or black-box ML models may not find clinical application on ethical grounds. The future of preeclampsia prediction using ML lies in balancing model performance with interpretability. Post hoc model agonistic explanation techniques like SHAP or LIME are promising tools that are increasingly being deployed. Human oversight remains indispensable in the implementation and interpretation of these models in clinical settings. Further research and validation across diverse populations especially the underrepresented regions like Africa and South Asia, where the burden of preeclampsia is significant, are critical to establishing the universal applicability of these promising ML-based approaches.
